# Anti-Inflammatory, Antioxidant, Chemical Characterization, and Safety Assessment of *Argania spinosa* Fruit Shell Extract from South-Western Morocco

**DOI:** 10.1155/2021/5536030

**Published:** 2021-08-03

**Authors:** Rachida Makbal, Fatima Ezzahra Janati Idrissi, Tarik Ouchbani, Maroua Ait Tastift, Hajar Kiai, Abdellatif Hafidi, Chemseddoha Gadhi

**Affiliations:** ^1^Laboratory of Agri-Food, Biotechnology and Valorization of Plant Resources, Phytochemistry and Pharmacology of Medicinal Plants Unit, Semlalia Faculty of Sciences, Cadi Ayyad University, P.O.B. 2390, 40000 Marrakech, Morocco; ^2^Laboratory of Extraction Processing and Natural Products Analysis, Agro-Food Industry Section, Hassan II Institute of Agronomy and Veterinary Medicine, P.O.B. 6202, Madinat Al Irfane, Rabat, Morocco

## Abstract

*Argania spinosa* (L.) plays an important role in the Moroccan agroeconomy, providing both employment and export revenue. Argan oil production generates different by-products with functionalities that are not yet investigated, in particular, the shell fruit. The present study aims, for the first time, at evaluating the acute and subacute toxicity, anti-inflammatory, and antioxidant effects of argan fruit shell ethanol extract (AFSEE). The LD_50_ of AFSEE was determined to be greater than the 5000 mg/kg body weight of mice. No significant variation in the body and organ weights was observed after 28 days of AFSEE treatment compared to that of the control group. Biochemical parameters and histopathological examination revealed no toxic effects of AFSEE. The AFSEE produced a significant inhibition of xylene-induced ear edema in mice. AFSEE reduced significantly the paw edema in mice after carrageenan injection. The chemical characterization showed that AFSEE contains a high level of total phenol content, flavonoids, condensed tannins, and flavanols. The obtained IC_50_ of DPPH, ABTS, reducing power, and *β*-carotene demonstrates that AFSEE has a potential antioxidant effect. The results indicate that AFSEE was safe and nontoxic to mice even at higher doses. Furthermore, the present findings demonstrate that AFSEE has potential anti-inflammatory and antioxidant activities.

## 1. Introduction

Medicinal plants have been used in folk medicine from ancient times due to their assumed acceptability, effectiveness, and low-cost free access for a large portion of the world's population [1]. Around 80% of the world's population relies on traditional medicine to treat their diseases, particularly in developing countries [2]. Recently, the consumption of natural herb formulations, used for thousands of years, has gained popularity in both developed and developing countries and widespread consideration as safer than chemical drugs for human health [3]. However, these natural products lack scientific data on their efficacy and safety and could subsequently lead to serious users' health damage due to their chemical complexity [4]. Therefore, the safety of natural products has been called into a question, through the assessment of the toxicity or harmful effects of many medicinal plants.

Argan tree (*Argania spinosa* (L.) Skeels) of the Sapotaceae family is an endemic and medicinal tree which exclusively grows in South-Western Morocco in an area of over 320000 square miles playing an essential function in the sustainable development of this part of the world. Moreover, argan tree supports indigenous populations economically since almonds are used to produce the well-known argan oil, which is largely used for cooking, in cosmetics, and for its various medicinal properties [5]. In fact, in Moroccan folk medicine, almost all the parts of *A. spinosa* are used for treating diseases. The ethnopharmacological study showed that argan oil is used against rheumatism and for healing burns. It is also used as an aphrodisiac and has spermatogenetic property. On the other hand, leaf infusion is drunk to treat gastritis, diarrhea, fever, and headaches. Argan fruit pulp is used to treat urticaria and dandruff but is mainly used for hide tanning. The almond paste is advised in case of flaky scalp, hair loss, eczema, and urticaria. The root of the tree is used for the treatment of diabetes and colopathies [6]. Meanwhile, argan fruit shell is simply used as fuel. Scientific reports have mainly focused on the validation of the biological properties of argan oil and some of its by-products.

Therefore, the aim of this study was at prospecting whether argan fruit shell ethanolic extract (AFSEE), which has never been investigated, possesses potential biological activities and may be considered to be safe for this purpose. The present study aimed at investigating the toxicity, anti-inflammatory, and antioxidant effects of argan fruit shell for the first time.

## 2. Materials and Methods

### 2.1. Identification and Collection of Plant Material

The fruits of *Argania spinosa* (L.) Skeels (family: Sapotaceae) were collected in June 2016 from Mirght (Sidi Ifni region) in the southwestern part of Morocco (29°25′14.46′′N; 9°42′38, 37′′O). The plant was identified by Prof. Ahmed Ouhammou, head of the Regional Herbarium MARK where a voucher specimen was deposited (MARK10888).

The pulps of argan fruits were manually removed, and then, the nuts were cracked to separate the almonds from the shells.

### 2.2. Animals and Ethics Statement

*In vivo* experiments were performed with adult *Swiss albino* male (8–10 weeks), weighing 28 ± 4 *g*, which were provided by the Animal Care Facility of the Faculty of Sciences Semlalia, Cadi Ayyad University, Marrakech, Morocco. Animals were maintained in a room at a controlled temperature of 22 ± 2°*C* and a 12-h light/dark cycle with free access to food and water. This animal study was conducted in accordance with the recommendations in the guidelines of the European Council Directive (EU2010/63) and met the ethical standards and approvals of the Council Committee of the Research Laboratories of the Faculty of Sciences, Cadi Ayyad University of Marrakech.

### 2.3. Preparation of Argan Fruit Shell Ethanol Extract (AFSEE)

The fruit shells of *A. spinosa* were dried and then finely grounded to powder (IKA WERE, M20, Germany). A quantity (500 g) of the powdered shell was extracted for two weeks in the dark with 5000 ml of ethanol (70%) at room temperature (25 ± 2°C). The mixture was filtered and the extract was concentrated to dryness under vacuum using a rotary evaporator (BUCHI Switzerland, Rotavapor R-210, Vacuum Pump V-700) at 40°C. The residue (3.5% *w*/*w*) was stored at −20°C until use.

### 2.4. Total Phenolic Content

Total phenolic content of tested extract (AFSEE 1 mg) was measured by spectrophotometer (model: VR-2000, no: 4120026, and wavelength rate: 325–1000 nm) at 725 nm using Folin–Ciocalteu reagents according to the method of Catalano et al. [4], using gallic acid as a standard. The total phenolic content of AFSEE was expressed as mg of gallic acid equivalents (GAE) per g of dry weight.

### 2.5. Total Flavonoid Content

Total flavonoid content of AFSEE (2 mg) was determined at 510 nm following the aluminum chloride colorimetric method of Zhishen et al. [7]. The total flavonoid content was expressed as mg of catechin equivalent (CAE) per g of dry weight.

### 2.6. Total Condensed Tannins

Condensed tannins were determined in AFSEE (4 mg) at 550 nm according to the method of Xu and Chang [8]. The amount of total condensed tannins was expressed as mg of CAE per g of dry weight.

### 2.7. Antioxidant and Radical Scavenging Activities

#### 2.7.1. Free Radical Scavenging Ability

The free radical scavenging capacity of AFSEE (0.0625, 0.125, 0.25, and 0.5 mg/ml) was evaluated at 517 nm using a stable 2,2-diphenyl-2-picrylhydrazyl radical (DPPH) according to the method of Von Gadow et al. [9]. The free radical scavenging capacities of butylated hydroxytoluene (BHT), quercetin, and vitamin C were also investigated and compared to AFSEE. The blank used is the ethanol.

The capacity antioxidant of the AFSEE against DPPH was calculated using the following equation:

(1) $$\begin{array}{c} \%\text{inhibition}=\left[\frac{A_{\text{control}}-A_{\text{sample}}}{A_{\text{control}}}\right]\times 100. \end{array}$$

#### 2.7.2. ABTS Radical Cation Decolorization Assay

The free radical scavenging activity of AFSEE (0.125, 0.25, 0.5, and 1 mg/ml) was also evaluated at 734 nm using ABTS (2,2′-azinobis-3-ethylbenzothiazoline-6-sulphonate) radical cation decolorization assay following the method of Li et al. [10]. Trolox was used as a standard and the capacity of free radical scavenging was expressed as mM trolox equivalents/ml. The blank used is the ethanol.

The capacity antioxidant of the AFSEE against ABTS was calculated using the following equation:

(2) $$\begin{array}{c} \%\text{inhibition}=\left[\frac{A_{\text{control}}-A_{\text{sample}}}{A_{\text{control}}}\right]\times 100. \end{array}$$

#### 2.7.3. Reducing Power Capacity

The ability of AFSEE (0.03125, 0.0625, 0.125, 0.25, and 0.5 mg/ml) to reduce Fe^3+^ at 700 nm was evaluated using the method described by Oyaizu [11]. BHT, quercetin, and vitamin C were used as positive controls. The blank used is the ethanol with reaction medium.

#### 2.7.4. *β*-Carotene Bleaching Assay

*β*-Carotene bleaching test was carried out according to the method reported by Kartal et al. [12] with some modifications. 1 ml of *β*-carotene/chloroform solution (0.2 mg/ml) was added to round-bottom flasks containing 25 *μ*l linoleic acid and 200 *μ*l Tween 20. Chloroform was evaporated at 40°C under vacuum and the resulting mixture was diluted with 100 ml of distilled water and was shacked well. Aliquots of 2.5 ml of this emulsion were added to a series of test tubes containing 350 *μ*l of different concentrations of tested extract, positive controls (BHT and quercetin), or negative controls (ethanol). The absorbance of the mixtures at 470 nm was immediately measured after 2 hours at 50°C. The blank used is the ethanol with reaction medium without *β*-carotene. The capacity of the AFSEE to protect *β*-carotene from oxidation was calculated using the following equation:

(3) $$\begin{array}{c} \%\text{bleaching inhibition}=\left[\frac{A_{0}-A_{1}}{A_{1}-A_{2}}\right]\times 100, \end{array}$$

where *A*_0_ is the absorbance of the sample at 120 min, *A*_1_ is the absorbance of the control at 0 min, and *A*_2_ is the absorbance of the control at 120 min.

All tests were performed in triplicate and the graph was plotted with the average of the three determinations.

### 2.8. In Vivo Toxicological Tests

#### 2.8.1. Acute Oral Toxicity

The acute toxicity test was performed according to the Organization for Economic Co-operation and Development (OECD) guideline N° 425 [13] where the limit test dose of 5000 mg/kg was used. Male mice were randomly selected and kept under standard conditions for five days. All animals were fasted for 12 h prior to administration. Each mouse from the treated groups was administered by the intragastric route, a single oral dose of AFSEE (2000 or 5000 mg/kg) dissolved in physiological water at 10 ml/kg, while the control group received physiological water (10 ml/kg). The animals were observed within the first 4 hours after administration and daily during 14 days to detect any signs of toxicity. Their general behavior and the number of deaths were recorded. Animals' body weight and their dietary intake were daily recorded during the 14 days of experiment. At the end of this period, all animals were sacrificed under ether anesthesia after a 12 h overnight fasting. The heart, lungs, spleen, liver, and kidney were removed and weighed. The relative organ weight of each animal was calculated as follows:

(4) $$\begin{array}{c} \text{Relative organ weight}=\left[\frac{\text{absolute organ weight }\left(\text{g}\right)}{\text{body weight of the animal on sacrifice day }\left(\text{g}\right)}\right]\times 100. \end{array}$$

#### 2.8.2. 28-Day Subacute Oral Toxicity

The 28-day repeated oral toxicity study was performed according to the OECD guideline 407 [14]. Based on results of acute toxicity and limit tests, animals were randomly divided into 5 groups of six mice. AFSEE-treated groups received daily, during the 28 days, an oral administration of AFSEE at 250, 500, 1000, and 2000 mg/kg body weight. The fifth group served as negative control and daily received physiological water (10 ml/kg). Animals were daily observed for any toxic signs and weighed, and their food intake was recorded. After 28 days, all surviving animals were fasted overnight and anesthetized with urethane (1 g/kg, i.p.). The blood samples were collected into nonheparinized tubes for separation of serum and biochemical analysis (aspartate aminotransferase (AST), alanine aminotransferase (ALT), creatinine, and urea). The vital organs of sacrificed mice were then removed and weighed (heart, liver, spleen, kidney, and lungs) to determine the relative organ weights and observed for any gross lesions. The organs were preserved in 10% buffered formaldehyde solution for histopathological study [15].

### 2.9. Anti-Inflammatory Activity

To assess the effect of AFSEE on acute inflammation, two *in vivo* tests, xylene-induced ear edema and carrageenan-induced paw edema, were performed. Adult *Swiss albino* male mice were randomly assigned to five groups of six animals. All animals were fasted 18 h before the test. The control group received physiological water (10 ml/kg), the test groups received different concentrations of AFSEE (125, 250, or 500 mg/kg), and the standard group received the reference drug (diclofenac, 25 mg/kg).

#### 2.9.1. Xylene-Induced Ear Edema

The xylene-induced ear edema test was carried out according to Tang et al.'s method [16]. The animals were orally administered with physiological water (negative control), AFSEE (125, 250, or 500 mg/kg), or the reference drug diclofenac (25 mg/kg). After the pretreatment, the acute inflammation was induced on the anterior and posterior surfaces of the right ear by a topical application of 20 *μ*l/ear of xylene. After one hour of xylene application, animals were sacrificed; both ears were removed and ear sections of a 6 mm diameter were punched out and weighed.

To evaluate the extent of the ear edema, the weight difference between the right and left ear sections of the same animal was measured. The inhibition percentage was calculated by the following equation:

(5) $$\begin{array}{c} \text{Inflammation rate}\%=\left(\frac{1-{\text{WE}}_{\text{left}}}{{\text{WE}}_{\text{right}}}\right), \end{array}$$

where WE_left_ and WE_right_ is the weight of left and right ear.

(6) $$\begin{array}{c} \text{Inflammation inhibition}\%=\left[\frac{\text{inflammation rat}{\text{e}}_{\text{control}}-\text{inflammation rat}{\text{e}}_{\text{sample}}}{\text{inflammation rat}{\text{e}}_{\text{control}}}\right]\times 100. \end{array}$$

#### 2.9.2. Carrageenan-Induced Mice Paw Edema

Carrageenan-induced paw inflammation in mice was performed according to the method described by Huang et al. [17]. One hour after the oral administration of AFSEE (125, 250, and 500 mg/kg) or the reference drug (diclofenac, 25 mg/kg), the carrageenan solution (0.02 ml of 2% carrageenan suspended in 0.9% NaCl) was subcutaneously injected into the plantar surface of the right hind paw of mice. The paw volume was measured using a digital caliper (vernier) at several time points: at 1 h, 2 h, 3 h, and 4 h after carrageenan administration. The anti-inflammatory activity was calculated as percentage inhibition of edema in the animals treated with extract under test in comparison to that in the carrageenan control group. The percentage inhibition of edema is calculated using the formula:

(7) $$\begin{array}{c} \text{Inflammation inhibition}\%=\left[\frac{T_{\text{control}}-T_{\text{sample}}}{T_{\text{control}}}\right]\times 100, \end{array}$$

where *T*_sample_ is the thickness of mice paw given test drug or extract at corresponding time and *T*_control_ is the paw mice thickness of the control group at the same time.

### 2.10. Statistical Analysis

Data were expressed as the mean values ± standard deviation (SD) for each measurement. The results were analyzed by one way analysis of variance (one-way ANOVA). Post hoc procedure was used for significance of difference (*p* < 0.05). Analysis was performed with SPSS 13.0 software version (SPSS Inc.; Chicago, IL, USA).

## 3. Results

### 3.1. Total Phenolic, Flavonoid, and Condensed Tannin Content Quantification in AFSEE

Total phenolic, flavonoid, and condensed tannin contents are presented in Table 1. Data indicated that AFSEE contains a high amount of polyphenols that is around 22.1 ± 0.87 mg gallic acid eq/g dw. Total flavonoids registered in AFSEE reached up to 9.9 ± 0.2 mg catechin eq/g dw, while the condensed tannin content was estimated to be 1.6 ± 0.08 mg catechin eq/g dw. Total flavanol found in AFSEE is around 2.4 ± 0.03 catechin eq/g dw.

### 3.2. Antioxidant Activity

AFSEE exhibited an interesting antioxidant with the four methods used (DPPH, ABTS, reducing power, and the *β*-carotene bleaching assay) compared with natural and commercial antioxidants. AFSEE was able to reduce the stable, purple-colored radical DPPH into yellow-colored DPPH-H with an IC_50_ value of 0.38 ± 0.005 mg/ml (Table 2). The obtained data also demonstrated that AFSEE exhibits a great scavenging potential against ABTS^**+**^ (IC_50_ = 8.23 ± 0.08 mM trolox equivalent/ml). Moreover, AFSEE revealed a good reducing power as well as a great antioxidant capacity against the bleaching of *β*-carotene induced by free radical, generated from linoleic acid oxidation, with IC_50_ values of 0.16 ± 0.004 mg/ml and 0.79 ± 0.008 mg/ml, respectively.

### 3.3. Acute Toxicity

#### 3.3.1. Acute Oral Toxicity

**Figure 1:**
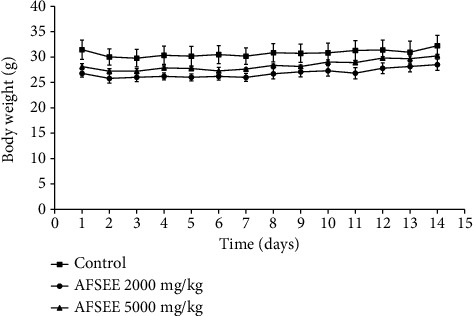
Evolution of the body weight of male mice treated with a single dose of AFSEE. The values are expressed as mean ± SD (*n* = 6). No significant difference was recorded among groups (*p* < 0.05 versus control group).

**Figure 2:**
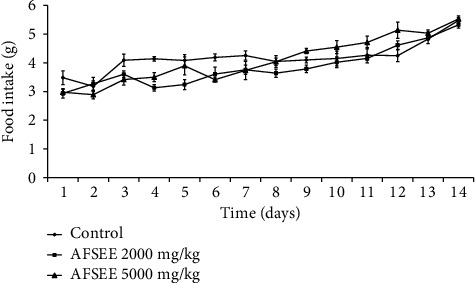
Food intake of male mice treated with a single dose of AFSEE. The values are expressed as mean ± SD (*n* = 6). No significant difference was recorded among groups (*p* < 0.05 versus the control group).

The oral administration of single doses (2000 and 5000 mg/kg body weight) of AFSEE did not induce any mortality among treated animals during the fourteen days of the assay. No signs of toxicity (diarrhea, hypoactivity, hyperventilation, motor impairment, sedation, piloerection, abdominal contortions, muscle tone, or convulsions) or significant body weight (Figure 1), dietary intake (Figure 2), or relative organ weight (Table 3) changes were observed among treated animals. These data show that oral lethal dose 50 (LD_50_) of AFSEE was much higher than 5000 mg/kg body weight of mice.

#### 3.3.2. 28-Day Repeated Oral Toxicity Study

**Figure 3:**
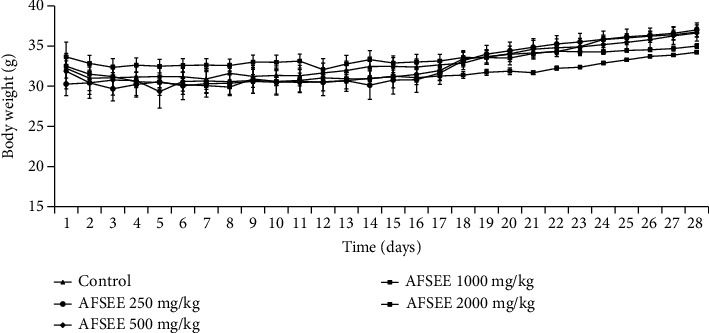
Body weight of male mice treated with or without AFSEE for 28 consecutive days. The values are expressed as mean ± SD (*n* = 6). No significant difference was recorded among groups (*p* < 0.05 versus the control group).

**Figure 4:**
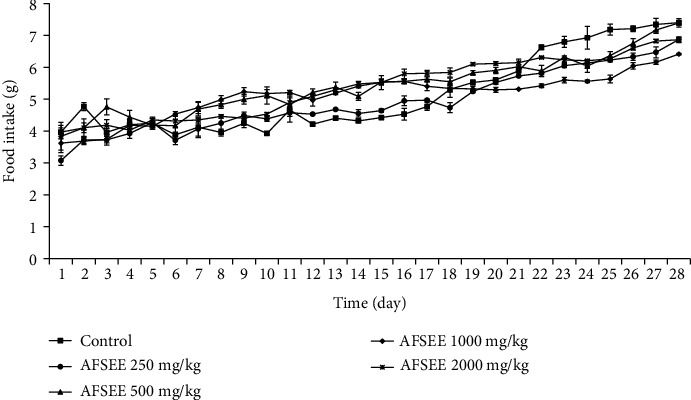
Food intake of male mice treated with or without AFSEE within 28 consecutive days. The values are expressed as mean ± SD (*n* = 6). No significant difference was recorded among groups (*p* < 0.05 versus the control group).

AFSEE subacute toxicological effects were carried out in mice orally treated with 125, 250, 500, 1000, and 2000 mg/kg of the extract during 28 days. Throughout this period, neither death nor significant alterations in mice behavior were recorded. The obtained results show that treatment with AFSEE (125, 250, 500, 1000, and 2000 mg/kg) did not affect the body weight (Figure 3), dietary intake (Figure 4), and the relative organ weight (Table 4) of mice when compared to control group, reaffirming the absence of toxicity of AFSEE in 28-day repeated oral exposition.

The biochemical analysis of serum transaminases AST (aspartate aminotransferase) and ALT (alanine aminotransferase) revealed that AFSEE treatment did not promote significant alterations in the serum transaminases (Table 5). Likewise, no change in the creatinine and urea levels was observed when compared to the control group. The data demonstrate that biochemical parameters remained within physiological range throughout the treatment period indicating that AFSEE did not affect the liver and kidney function.

**Figure 5:**
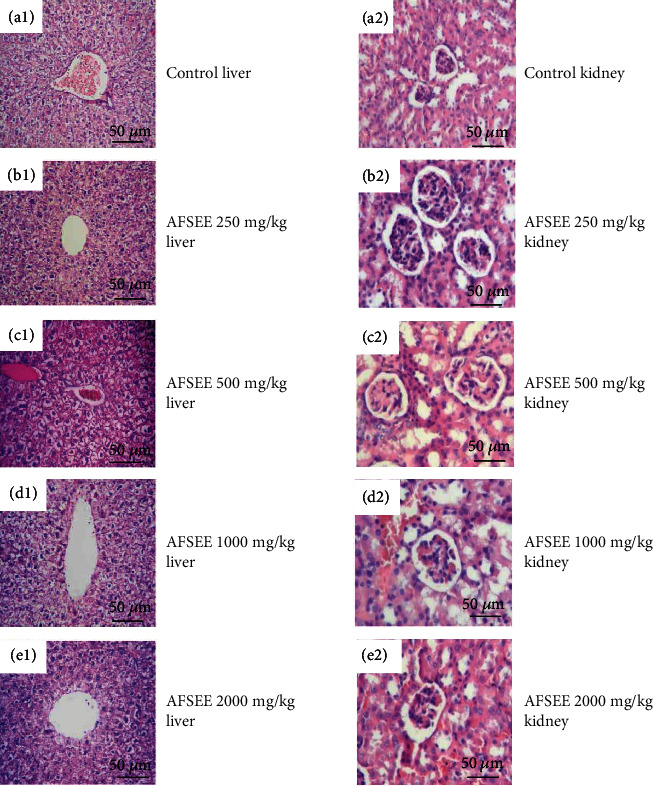
Histopathological analyses of the liver and kidney in male mice after oral administration for 28 days. Liver: A1 (control HE, 200x), B1 (AFSEE 250 mg/kg HE, 200x), C1 (AFSEE 500 mg/kg HE, 200x), D1 (AFSEE 1000 mg/kg HE, 200x), and E1 (AFSEE 2000 mg/kg HE, 200x). Kidney: A2 (control HE, 200x), B2 (AFSEE 250 mg/kg HE, 200x), C2 (AFSEE 500 mg/kg HE, 200x), D2 (AFSEE 1000 mg/kg HE, 200x), and E2 (AFSEE 2000 mg/kg HE, 200x). Sections of liver and kidney showing no significant histopathological changes after administration of AFSEE for 28 days compared to the control group (*n* = 6 each group).

The macroscopic analysis of the liver and kidney from animals treated with AFSEE for 28 days revealed no abnormalities in the color or texture in comparison to those from the control group. The histopathology analysis of the liver and kidney was carried out, and corroborating with macroscopic analysis, the results shown in Figure 5 demonstrate the normal aspect of liver and kidney cells, without degenerative lesions.

### 3.4. Anti-Inflammatory Effect of AFSEE

#### 3.4.1. Xylene-Induced Ear Edema

The average weight of the ears and the percentage inhibition of inflammatory response are presented in Table 4. The ear edema was measured as weight increase of the right ear over the left one. The results showed that after a topical application of xylene on the ear, the difference of ear weight in the control group was significantly higher compared to those in treated groups (Table 6). In fact, AFSEE at doses of 125 (33.88%), 250 (36.82%), and 500 (65.6%) mg/kg body weight significantly inhibited the xylene-induced ear edema in mice (for all, *p* < 0.05 versus the control group) with high effect at a highest dose 500 mg/kg body weight. Diclofenac (25 mg/ml/ear) exhibits a considerable anti-inflammatory activity by reducing the ear edema by 71.11%. The data suggest that AFSEE could obviously inhibit the ear edema induced by xylene in a dose-dependent manner (Table 6).

#### 3.4.2. Effect of AFSEE on Carrageenan-Induced Mouse Paw Edema

The anti-inflammatory activity of AFSEE against paw edema was assessed and the obtained results were shown in Table 7. After the carrageenan treatment, the paw volume of the carrageenan control group obviously increased in a time-dependent manner. However, the paw volume of animals treated with AFSEE 125 (19.45%), 250 (22.10%), and 500 (21.10%) mg/kg body weight decreases significantly from the 2^nd^ to the 4^th^ hour, following carrageenan treatment (*p* < 0.05) (Table 7) with a great effect at a high doses of 250 and 500 mg/kg body weight. The tested doses of AFSEE exhibit maximum anti-inflammatory activity approximately at the 4^th^ hour (for all, *p* < 0.05 versus the carrageenan control group). Oral administration of AFSEE at the dose of 500 mg/kg has the same anti-inflammatory activity as diclofenac (17.68%) (25 mg/kg body weight).

## 4. Discussion

Since time immemorial, people widely used medicinal plants to prevent and treat diseases, considering them as safe and effective treatment due to their low cost and negligible side effects. In fact, the medicinal plants could provide valuable pharmacological activities due to their bioactive compounds [18]. However, the random use of these plants without taking into account their possible toxicity may lead to serious health problems.

*Argania spinosa* is one of the most widely used medicinal plants in the south Moroccan region. Despite the interesting properties of argan tree by-products, frequently used in folk medicine, their toxic effects have seldom been investigated. There is no study regarding the pharmacological and toxicological effects of argan fruit shell, which represents 55% of the dry weight of the argan fruit [19]. In this context, we investigated the acute and subchronic toxicity, the anti-inflammatory, and the antioxidant activities of AFSEE using *in vitro* and *in vivo* experimental models.

The result of acute toxicity showed that oral administration of AFSEE at a single dose (2000 and 5000 mg/kg body weight) did not cause mortality in mice, since the animals survived until the sacrifice day. Likewise, the growth as well as the relative organ weight of treated animals with AFSEE was not affected throughout the experimental period. Therefore, the LD50 of AFSEE was higher than 5000 mg/kg.

Regarding the evaluation of the oral subacute toxicity, carried out at doses of 250, 500, 1000, and 2000 mg/kg for 28 days, no mortality was observed. Similarly, AFSEE had no effect on the growth and functions of mice at these doses within this period of treatment.

To strengthen those results, biochemical markers of liver and kidney function were performed. Our data revealed that 28-days of repeated administration of AFSEE at different doses did not promote significant alterations in the level of serum transaminases (AST and ALT). It was reported that transaminases are important indicators of hepatic damage and any increase in their serum level signals hepatic injury [20, 21]. On the other side, kidney functions were assessed by creatinine and urea analyses. The obtained results demonstrated that the tested doses of AFSEE did not induce any modification in creatinine and urea levels, showing that AFSEE did not favor renal alterations. Indeed, the increase in their levels is a strong indicator of renal dysfunction [22].

The absence of a hepatorenal toxicity induced by AFSEE was confirmed by the histological examination of the liver and the kidney. Animals treated with AFSEE for 28 days at various doses have no toxicological signs in liver and kidney tissues. Indeed, no sinusoids, apoptotic nuclei, or inflammatory infiltrate inside sinusoidal capillaries in the liver were observed. Likely, no disorganization of tubules and glomeruli and enlarged interstitial spaces in kidney were noticed. In addition to histological analysis, no difference was noticed in the organ weight. Thus, the data demonstrated that AFSEE did not induce either hepatotoxicity or nephrotoxicity in animals treated with repeated doses. Therefore, the extract may be classified as nontoxic [23]. The fruit of *A. spinosa* was originally used to feed livestock [6].

Two common inflammatory models to evaluate the anti-inflammatory activity of AFSEE were used: the xylene-induced ear edema and the carrageenan-induced paw edema in mice [24, 25]. Xylene induces various inflammatory responses including edema formation [26]. After the topical application of xylene, the interstitial fluid volume increases, resulting from the fluid shift from the intravascular to the interstitial compartment [27]. In addition, the ear edema induced by xylene involves inflammatory mediators such as histamine, kinin, and fibrinolysin. These mediators can promote vasodilation, increase vascular permeability, and cause edematous changes of the skin partially related with phospholipase A2 [28].

The second inflammatory model used was carrageenan. Carrageenan includes sulphated sugars that activate the complement system and the inflammatory mediators [29]. Postcapillary venules are dilated by carrageenan, leading to pathological exudation of inflammatory mediators and immune cells. The release of acute-phase inflammatory mediators takes place as the first step in carrageenan-induced paw edema [30].

The obtained results showed that AFSEE reduced significantly xylene-induced ear edema in a dose-dependent manner and exhibited a moderate inhibitory effect at the early phase in carrageenan-induced paw edema. However, at the late phase AFSEE presented a significant anti-inflammatory activity. The anti-inflammatory activity of AFSEE might be due to its high content in polyphenols particularly flavonoids and flavanols previously determined. Those compounds were reported to have a great power of reducing the edema induced by xylene and carrageenan agents [31].

The secondary metabolites mainly flavonoids might be responsible individually or in synergy at least in part, on the anti-inflammatory effect of AFSEE. The anti-inflammatory action of flavonoids is caused by the inhibition of enzymes such as cyclooxygenase (COX) and lipoxygenase and the inhibition of inflammatory mediators thereby decreasing inflammatory mediators such as prostaglandins and leukotrienes [32, 33]. It has been found that flavonoids inhibit the production and the release of histamine and inflammatory substances, probably by stabilizing cell membranes of mast cells [34, 35]. Flavonoids also inhibited the development of the induced granuloma [36]. On the other hand, condensed tannins are considered as antagonists of specific hormone receptors or inhibitors of some enzymes such as COX enzymes [37]. In addition, the flavanols share the capability to effectively suppress the production of cytokines that promote inflammation [38].

In the present work, we also proved the antioxidant potency of AFSEE through the DPPH, ABTS, reducing power, and *β*-carotene bleaching methods. This effect may be due to its content in polyphenols. This funding corroborates with the data previously reported. Indeed, it has been reported that the shell fruit of *A. spinosa* has a great antioxidant effect due to phytochemical constituents: procyanidin B1; B2, (−)−epicatechin, isoquercitrin, hyperoside, rutin, phloridzin, and myricetin [39, 40].

The present study revealed that AFSEE has a potential anti-inflammatory effect and antioxidant capacity. Taken together, we suggest that the anti-inflammatory effect might be attributed to the antiradical potential of AFSEE compounds. However, further studies are needed to underline the action mechanism of AFSEE extract and identify the molecules contributing to its biological effect.

## 5. Conclusion

In conclusion, these findings demonstrate for the first time that oral administration of AFSEE at up to 5000 mg/kg once or up to 2000 mg/kg body weight/day for 28 days did not cause either mortality or toxicity of animals. In addition, the present work clearly shows that AFSEE displayed an excellent effect against acute inflammation (xylene-induced ear edema and carrageenan-induced paw edema) in a dose-dependent manner and a potential antioxidant activity. Thus, the obtained results suggest that the argan fruit shell can be considered as a potential source of low cost and available highly added value products that can be used in pharmaceutical and food industries.

## Figures and Tables

**Table 1:** Mean values of total polyphenol, flavonoid, tannin, and flavanol contents in AFSEE.

AFSEE content			
Total polyphenols	Total flavonoids	Total tannins	Total flavanols
(mg GAE/g dw)	(mg CAE/g dw)	(mg CAE/g dw)	(mg CAE/g dw)
22.1 ± 0.87	9.9 ± 0.2	1.6 ± 0.08	2.4 ± 0.03

Each value is represented as mean ± SD (*n* = 3) DW: dry weight; GAE: gallic acid equivalent; CAE: catechin equivalent.

**Table 2:** Antioxidant activities, expressed as IC_50_ values using DPPH, ABTS, reducing power, and *β*-carotene assays, for AFSEE, vitamin C, quercetin, and BHT.

	IC_50_			
	DPPH (mg/ml)	ABTS (mM trolox equivalent/ml)	Reducing power (mg/ml)	*β*-Carotene (mg/ml)
AFSEE	0.38 ± 0.01^d^	8.23 ± 0.08^d^	0.16 ± 0.01^c^	0.79 ± 0.01^c^
Vitamin C	0.14 ± 0.01^b^	1.15 ± 0.01^b^	0.052 ± 0.01^b^	ND
Quercetin	0.10 ± 0.01^a^	0.44 ± 0.01^a^	0.05 ± 0.01^b^	0.085 ± 0.01^b^
BHT	0.27 ± 0.01^c^	5.84 ± 0.03^c^	0.028 ± 0.01^a^	0.031 ± 0.01^a^

Values represent mean ± SD (*n* = 3). Values with different letters in the same column are significantly (*p* < 0.05) different. ND: not determined.

**Table 3:** The relative organ weight of male mice treated with single oral doses of AFSEE.

Organ	Relative organ weight (g/100 g bw)		
	Control	AFSEE (mg/kg)	
		2000	5000
Heart	0.45 ± 0.04^b^	0.47 ± 0.03^b^	0.45 ± 0.02^b^
Lungs	0.76 ± 0.03^d^	0.82 ± 0.03^d^	0.8 ± 0.03^d^
Liver	4.71 ± 0.3^f^	5.28 ± 0.22^f^	4.88 ± 0.11^f^
Spleen	0.4 ± 0.03^a^	0.43 ± 0.02^a^	0.42 ± 0.02^a^
Right kidney	0.71 ± 0.04^c^	0.74 ± 0.05^c^	0.69 ± 0.02^c^
Left kidney	0.71 ± 0.05^c^	0.73 ± 0.04^c^	0.69 ± 0.01^c^
Stomach	1.25 ± 0.03^e^	1.28 ± 0.06^e^	1.3 ± 0.05^e^

Each value is represented as mean ± SD (*n* = 6). bw: body weight. Means within rows bearing different letter superscripts ^a,b,c,d,e,^ and ^f^ are significantly different (*p* < 0.05 compared to the control group).

**Table 4:** The relative organ weight of mice treated daily with different doses of AFSEE during 28 consecutive days.

Organ		Relative organ weight (g/100 g bw)			
	Control	AFSEE (mg/kg)			
		250	500	1000	2000
Heart	0.46 ± 0.02^a^	0.48 ± 0.03^a^	0.45 ± 0.03^a^	0.45 ± 0.03^a^	0.47 ± 0.03^a^
Lungs	0.6 ± 0.02^e^	0.57 ± 0.01^e^	0.58 ± 0.02^e^	0.64 ± 0.04^e^	0.61 ± 0.02^e^
Liver	3.94 ± 0.14^g^	3.77 ± 0.15^g^	3.99 ± 0.14^g^	3.9 ± 0.28^g^	4.20 ± 0.21^g^
Spleen	0.5 ± 0.03^d^	0.54 ± 0.27^d^	0.57 ± 0.49^d^	0.58 ± 0.22^d^	0.49 ± 0.02^d^
Right kidney	0.56 ± 0.01^b^	0.56 ± 0.04^b^	0.54 ± 0.04^b^	0.54 ± 0.01^b^	0.64 ± 0.02^b^
Left kidney	0.56 ± 0.02^c^	0.59 ± 0.03^c^	0.56 ± 0.02^c^	0.55 ± 0.01^c^	0.63 ± 0.02^c^
Stomach	0.75 ± 0.02^f^	0.83 ± 0.01^f^	0.85 ± 0.08^f^	0.81 ± 0.03^f^	0.79 ± 0.05^f^

Each value is represented as mean ± SD (*n* = 6). bw: body weight. Means within rows bearing different letter superscripts ^a,b,c,d,e,f,^ and ^g^ are significantly different (*p* < 0.05 compared to the control group).

**Table 5:** Effect of AFSEE on blood biochemical parameters of mice treated orally for 28 consecutive days.

Parameter	Unit	Control	AFSEE (mg/kg bw)			
			250	500	1000	2000
Aspartate aminotransferase (AST)	U/l	73.96 ± 3.08^a^	75.63 ± 2.06^a^	80.07 ± 4.80^a^	74.9 ± 3.21^a^	73.70 ± 0.70^a^
Alanine aminotransferase (ALT)	U/l	50.00 ± 7.66^b^	42.47 ± 0.95^b^	44.53 ± 2.61^b^	46.83 ± 1.53^b^	45.63 ± 1.46^b^
Creatine	Mg/dl	0.27 ± 0.03^c^	0.24 ± 0.02^c^	0.27 ± 0.02^c^	0.29 ± 0.05^c^	0.32 ± 0.03^c^
Urea	g/l	0.35 ± 0.05^d^	0.44 ± 0.08^d^	0.38 ± 0.03^d^	0.45 ± 0.03^d^	0.42 ± 0.04^d^

Each value is represented as mean ± SD (*n* = 6). bw: body weight. Means within rows bearing different letter superscripts ^a,b,c,^ and ^d^ are significantly different (*p* < 0.05 compared to the control group).

**Table 6:** Effect of AFSEE (125, 250, and 500 mg/kg bw) and diclofenac on xylene-induced ear edema in mice.

Treatment	Dose (mg/kg bw)	Ear edema (mg)	Inhibition (%)
Control	0	45.33 ± 0.56^d^	—
Diclofenac	25	13.10 ± 0.67^a^	71.11 ± 1.5^a^
AFSEE	125	31.37 ± 0.84^c^	30.80 ± 3.01^b^
	250	29.86 ± 0.52^b^	34.12 ± 1.87^b^
	500	15.60 ± 0.93^a^	65.6 ± 2.06^a^

Each value is represented as mean ± SD (*n* = 6). bw: body weight. Values with different letter superscripts ^a,b,^ and ^c^ in the same column are significantly (*p* < 0.05 compared to the control group) different.

**Table 7:** Effect of AFSEE (125, 250, and 500 mg/kg bw) and diclofenac (25 mg/kg) on carrageenan-induced paw edema in mice.

Treatment		Mean paw volume ± SD (ml) (% inhibition)				
		0 h	1 h	2 h	3 h	4 h
Control		2.74 ± 0.22^a^	3.47 ± 0.11^b^	3.32 ± 0.06^b^	3.10 ± 0.03^b^	3.02 ± 0.02^b^
Diclofenac (25 mg/kg bw)	Paw volume	3.2 ± 0.08^a^	2.95 ± 0.03^a^	2.7 ± 0.08^a^	2.58 ± 0.08^a^	2.48 ± 0.08^a^
	Inhibition	7.69%	14.9%	18.59%	16.67%	17.68%
AFSEE 125 mg/kg bw	Paw volume	3.83 ± 0.09^a^	3.33 ± 0.07^b^	3.00 ± 0.05^a^	2.65 ± 0.05^a^	2.43 ± 0.05^a^
	Inhibition	−10.48%	3.94%	9.55%	14.52%	19.45%
AFSEE 250 mg/kg bw	Paw volume	3.6 ± 0.11^a^	3.11 ± 0.1^ab^	2.83 ± 0.06^a^	2.65 ± 0.06^a^	2.35 ± 0.06^a^
	Inhibition	−3.85%	10.20%	14.67%	14.52%	22.10%
AFSEE 500 mg/kg bw	Paw volume	3.5 ± 0.04^a^	3.1 ± 0.03^ab^	2.92 ± 0.02^a^	2.75 ± 0.03^a^	2.38 ± 0.02^a^
	Inhibition	−0.96%	10.58%	11.96%	11.29%	21.10%

Each value is represented as mean ± SD (*n* = 6). bw: body weight. Values with different letter superscripts ^a^ and ^b^ in the same column are significantly (*p* < 0.05 compared to the control group) different.

## Data Availability

No data were used to support this study.
